# Changing Times Together? A Time‐Diary Analysis of Family Time in the Digital Age in the United Kingdom

**DOI:** 10.1111/jomf.12564

**Published:** 2019-03-11

**Authors:** Killian Mullan, Stella Chatzitheochari

**Affiliations:** ^1^ University of Oxford; ^2^ University of Warwick

**Keywords:** adolescence, childhood, children, families, family research, technology

## Abstract

**Objective:** This study examines the impact of digital mobile devices on different aspects of family time in the United Kingdom.

**Background:** Recent years have witnessed increasing concerns surrounding the consequences of the widespread diffusion of Internet‐enabled mobile devices such as smartphones for family well‐being. However, research examining the extent to which mobile devices have influenced family time remains limited.

**Method:** Using nationally representative time‐diary data spanning a period of unprecedented technological change (U.K. 2000 and 2015 Time Use Surveys), the authors construct a set of novel family time measures that capture varying degrees of family togetherness and examine changes in these measures over time. Novel diary data are also analyzed to explore the occurrence of mobile device use during different aspects of family time in 2015.

**Results:** Children and parents spent more time at the same location in 2015, and there was no change in the time they spent doing activities together. However, there was a marked increase of alone‐together time, when children were at the same location as their parents, but did not report that they were copresent with them. The results show that children and parents used mobile devices during all aspects of family time in 2015, but device use was notably concentrated during alone‐together time.

**Conclusion:** This study provides an empirical basis for documenting the impact of mobile device use on family time.

The past decade has witnessed an unprecedented diffusion of mobile devices such as smartphones and tablets in advanced economies, alongside the arrival of powerful Internet connections. According to research by the Pew Research Center, approximately three quarters of American adults own a smartphone (Poushter, 2016), with one in four reporting going online “constantly,” and 43% several times a day (Perrin & Jiang, 2018). Similarly, more than two thirds of adults and children in the United Kingdom own a smartphone, whereas one in two households own at least one tablet (Ofcom, 2015). Alongside these trends, academic and public debates around the perils of technology and ubiquitous connectivity for individual and societal well‐being have been increasing (Palmer, 2015; Turkle, 2011; Twenge, 2017; Wellman & Haythornthwaite, 2008). However, despite the fact that family scholars have long identified technological change as one of the key developments likely to influence family functioning (Daly, 1996; Ogburn & Nimkoff, 1955; Seltzer et al., 2005), research on the impact of contemporary information and communication technologies on family life remains limited (Carvalho, Francisco, & Relvas, 2015; Lanigan, 2009; Sharaievska, 2017). A number of studies have examined Internet or mobile device usage in relation to different family outcomes (Carvalho et al., 2015). The results are mixed, with some studies underlining a positive role of Internet or mobile devices in facilitating the communication and coordination of activities of family members (Chesley & Fox, 2012; Kennedy & Wellman, 2007; Ling, 2012), and others revealing negative effects for family relationships and the perceptions of family cohesion (Mesch, 2006; Sharaievska & Stodolska, 2017). However, we still lack evidence on the extent to which the advent of mobile devices such as smartphones has altered time allocation and daily activities in the family context. As a result, propositions surrounding the emergence of solitary device use and individualized screen‐based media consumption within the family home remain largely untested (Daly, 1996; Livingstone, 2002).

This study assesses the impact of mobile digital devices on family time by analyzing data from the U.K. 2000 to 2001 Time Use Survey (UKTUS 2000; Ipsos‐RSL, Office for National Statistics, 2003) and 2014 to 2015 Time Use Survey (UKTUS 2015; Gershuny & Sullivan, 2017). Using matched diary data of children and parents, we propose a novel set of family time measures, moving beyond existing studies that solely rely on parental diary records to construct a unidimensional measure of family time (Genadek, Flood, & Roman, 2016; Neilson & Stanfors, 2017). An innovative approach that prioritizes children's perspectives and encapsulates varying degrees of family togetherness is applied, allowing us to test propositions around the emergence of alone‐together time, attributed to the use of mobile devices and characterized by a lack of close interaction among family members (Turkle, 2011). Spanning a period of unprecedented diffusion of mobile devices among adults and children in the United Kingdom (Ofcom, 2015), the data allow us to assess the extent to which changes in different types of family time including alone‐together time are associated with recent technological change, controlling for other factors. A unique strength of our data is the availability of contextual diary information on mobile device use in the 2014 to 2015 survey that measures the concurrent use of mobile device use during other activities, which cannot be accurately captured by “stylized” time‐use questions in conventional social surveys (Hampton, 2017). This provides us with nationally representative evidence on the way mobile device use is woven into family life in contemporary Britain, allowing us to better understand trends in family time between 2000 and 2015. To our knowledge, ours is the first study that bridges insights from time‐diary research and literature on the effects of mobile device use, placing technological change in the foreground of sociological accounts of family time.

## Mobile Devices Diminishing Family Time?

Although there is no agreed definition of family time, fundamental to its conceptualization is the notion that it encapsulates different activities with varying levels of interaction among family members (Daly, 1996). This can range from time spent in shared meals or leisure activities deliberately aimed to foster a sense of family togetherness, to relatively unnoticed time as family members go about their daily routines (Christensen, James, & Jenks, 2000), what Daly, (1996) referred to as the “coincidental sharing of space and time that arises from the intersection of busy lives” (p. 67).

Earlier theoretical accounts identified a core tension surrounding the influence of technology on family time in that it can bring family members together while keeping them apart (Daly, 1996). Indeed, technology opens up opportunities for shared leisure activities, especially within the home, but it also provides room for family members to engage in separate individualized activities while being together at the same location. For example, although technologies such as cell phones and home computers have blurred the spatial boundaries between work and family, allowing parents to work from home and spend more time with children, they have also been shown to negatively affect family life by enabling constant contact with the workplace and distracting parents' attention (Blair‐Loy, 2009; Chesley, 2005). Similarly, home entertainment offers opportunities for numerous social activities within the home, but it may also lead to a “bedroom culture,” characterized by private video‐gaming and media consumption in different rooms of the household (Livingstone, 2002).

It can be argued that this tension became stronger following the advent of mobile devices that radically widened our access to information, entertainment, and social networks, entirely disconnecting it from the bindings of place and time. Rather than being shared household utilities such as the television or the fixed telephone line, mobile devices are tied to specific individuals (Thulin & Vilhelmson, 2007), thus offering unprecedented opportunities for individualized screen‐based activities at the expense of family time of closer interaction. The social implications of this have been problematized by several scholars such as Gergen (2002) who earlier coined the term *absent presence* to describe the new era of face‐to‐face interactions following the arrival of cell phones, arguing that geographical proximity is no longer important and that family members lead psychologically separate lives. In a similar manner, the renowned alone together thesis of Turkle (2011) postulated that, in the era of ubiquitous connectivity, people are increasingly using computers and mobile devices to interact with others while physically alone and are engaging in activities such as texting instead of closely interacting with those with whom they are physically copresent.

A plethora of qualitative studies have underlined the extent to which mobile devices are now embedded in family life (Bond, 2014; Clark, 2013; Ling, 2012; Thompson, Berriman, & Bragg, 2018). Several studies examining the contribution of Internet or mobile device use on different family outcomes have suggested that associations with family conflict and with perceptions of decreased family time and interactions among family members (Lee, 2009; Lee & Chae, 2007; Mesch, 2006; Nie, 2001). This is potentially a result of the negative effects of constant interruptions from mobile devices on the ways face‐to‐face interactions and shared activities are experienced (Kildare & Middlemiss, 2017; Misra, Cheng, Genevie, & Yuan, 2016; Przybylski & Weinstein, 2013; Thornton, Faires, Robbins, & Rollins, 2014).

In addition, research suggests that mobile devices have influenced important shared family activities such as families eating together and television viewing. For example, Radesky et al. (2014) documented caregivers' degree of absorption in mobile devices during family meals at fast‐food restaurants, which appeared to interrupt their interactions with children. Likewise, research suggests that mobile device use influences the nature and experience of television viewing within the home, as it takes place while family members watch television together (Holz, Bentley, Church, & Patel, 2015; Müller, Gove, Webb, & Cheang, 2015).

Taken together, these findings lend some support to academic and public concerns around the negative effects of mobile devices on family time and face‐to‐face interactions (Gergen, 2002; Turkle, 2011). However, this body of research is predominantly concerned with question about qualitative changes in these domains. In contrast with the abovementioned studies, time‐diary research suggests that family time has increased: For example, using U.S. data, Genadek et al. (2016) showed that the time parents spent together with their children increased steadily from 1965 through 2012. Neilson and Stanfors (2017) showed similar increases in the time parents report spending with children for Sweden using data from 1990 to 2010. Similarly, Sandberg and Hofferth (2001) found increases in the time children spend with parents, albeit for a period prior to the advent of mobile devices (1981–1997).

This body of research has not explicitly addressed questions about the impact of the widespread diffusion of mobile devices on family time. Instead, it has predominantly focused on long‐term trends and their association with changes in marriage, maternal employment, gender roles, and ideologies about parenting and family life (Genadek et al., 2016; Milkie, Mattingly, Nomaguchi, Bianchi, & Robinson, 2004; Neilson & Stanfors, 2017; Sayer, Bianchi, & Robinson, 2004). Employing a unidimensional family time measure on the total time parents are copresent with children, this research does not capture changes in the time family members are together but are not closely interacting as well as changes in time spent in shared activities, potentially as a result of mobile device use.

The first aim of our study is, therefore, to improve understandings of the influence of mobile devices on family time by analyzing change in different aspects of family time. Drawing on insights from existing literature on the influence of mobile devices on family life we construct a set of measures capturing different levels of family togetherness using information provided by both children and parents. For this investigation, we use U.K. data from 2000 and 2015, covering two time points before and after the widespread diffusion of Internet‐enabled mobile devices in the country.

## Mobile Devices Taking Over Family Time?

Public concerns about the influence of mobile devices on family time largely stem from the widespread view that both parents and children are spending excessive amounts of time using these devices. Qualitative research has been at the forefront in showing how mobile devices are now firmly embedded in family life (Bond, 2014; Clark, 2013; Ling, 2012; Thompson et al., 2018). However, there is little generalizable evidence on the amount of time family members spend using mobile devices. To a large extent, this is arguably a result of the methodological difficulty in obtaining valid measures of contemporary mobile device use with the use of traditional questionnaire approaches (Hampton, 2017). This relates to the nature and frequency of mobile device use in the era of perpetual connectivity, clearly captured by the shift from the phrases “going online” to that of “being online” (Williams & Merten, 2011, p. 150), which render “stylized” survey questions about time spent using mobile devices prone to recall and calculation difficulties and to varying understandings in what really constitutes mobile device use (Hampton, 2017; Nie, 2001; Robinson & Godbey, 2010; Vandewater & Lee, 2009).

For example, a recent nationally representative study examining use of digital devices of U.S. families asked parents the following question: “To the best of your knowledge, how much time did your child spend doing each of the following [using s computer; using a smartphone; using a tablet…] yesterday?” (Lauricella et al., 2016, p. 37) Notwithstanding the methodological problems noted previously, this question is also prone to social desirability bias as it cannot accurately capture device use that takes place concurrently with other family activities. In contrast, time‐diary estimates can provide a comprehensive account of the entire range of daily activities and their context, enabling empirical examinations of the ways contemporary mobile device use is associated with different types of time and activities, whereas they have also been evidenced to produce the most accurate and reliable measures of time use and daily behavior (Robinson & Godbey, 2010). An earlier study focusing on the media use of children and young people in the United States successfully employed the time‐diary methodology to demonstrate the frequent occurrence of using different media and devices at the same time (Rideout, Foehr, & Roberts, 2010). However, this research does not examine the usage of mobile devices during different types of family time and everyday activities.

We argue that, in addition to studying changes in family time, a further way to approach the question of the influence of recent technological changes on family time is to examine the extent to which mobile devices are “present” in the everyday lives of contemporary families. The second aim of our study is, therefore, to provide the first nationally representative study of the extent to which mobile device use is embedded in difference aspects of family time in the United Kingdom using novel diary data collected in 2015. Specifically, we analyze the time both children and parents spend using mobile devices during different types of family time, corresponding to differing levels of interaction, throughout the day. This complements our analysis of change in family time by providing further insights surrounding the influence of recent technological change on family life in Britain.

## Family Time, Mobile Devices, and Child Age

In analyzing both changes in family time and time using mobile devices, it is important to single out child age as a key factor as it is strongly associated with family time and mobile device use. It is well established that older children spend less time with their parents as they seek more independence (Crosnoe & Trinitapoli, 2008; Lam, McHale, & Crouter, 2012; Larson & Richards, 1991; Steinberg, 2002). In contrast, children's use of mobile devices is positively associated with age (Mullan, 2017), and older children are more likely to own mobile devices such as smartphones (Mascheroni & Olafsson, 2014). We may therefore expect older children to spend less time with family and more time using devices than younger children. It follows that any influence of mobile devices on family time may be concentrated among older children rather than younger children. For this reason, the age of the child constitutes an additional layer across our analyses, allowing us to provide a nuanced account of the influence of mobile devices in families with children of different ages.

## Method

### *Data and Samples*

We use data from the UKTUS 2000 and UKTUS 2015. The two surveys employed similar sampling and coding procedures, time‐diary instruments, and instructions to respondents to produce valid estimates of changes in time use in the United Kingdom. Both surveys obtained nationally representative samples of households and individuals using clustered, stratified sampling designs, and asked respondents to complete time diaries for two randomly allocated days, one weekday and one weekend day. A self‐completed, open‐ended, 24‐hour, time‐diary format was used in both surveys, with all members of selected households aged 8 years old and older describing their main and secondary activities in each 10‐minute slot and providing information on their location and with whom they were copresent.

The main difference between the two surveys is that UKTUS 2015 respondents were additionally provided with a diary column to record when they were using digital devices (smartphones, tablets, or computers) during the day. The lack of similar information in UKTUS 2000 does not allow us to measure change over time, and our analysis of mobile device use remains solely cross‐sectional.

We focus on a sample of children aged 8 to 16 years living in lone‐parent and heterosexual two‐parent households. Diary data from children and parents are matched to create our measures of family time (see later), and we therefore exclude children who are not coresident with a parent, including those living with their grandparent(s) (*n* = 14) or in other living arrangements (*n* = 32), from our analysis. We also drop 12 children because their parents did not complete any diaries, and we exclude one diary from a further 31 children because their parents completed one diary only. Our multivariate analysis controls for parental characteristics, and we exclude 43 children whose parents did not provide information on these characteristics in the survey questionnaire. As our family‐time measures also rely on children's copresence reports (see later), we restrict analyses to those diaries with less than 4 hours of missing copresence information, dropping 228 child diaries from the sample. The final sample for the analysis of change between 2000 and 2015 contains 4,993 diary days (2,558 children–parent pairs). For the analysis of mobile device use, we additionally exclude 36 diaries with 4 or more hours of unreported device use, yielding a sample of 1,775 diary days (932 children–parent pairs).

### *Measures of Family Time*

We construct a set of measures that represent varying degrees of family togetherness. Our broadest measure refers to the total time children and parents spend together at the same location, excluding time during which children or parents are asleep. We refer to this measure as *total family time*. Total family time captures an important spatial dimension that cuts across different aspects of family time (Christensen et al., 2000; Daly, 1996) and is constructed by using location information provided independently by children and parents in their time diaries. We note that, except where children explicitly report being copresent with a parent, if children or parents reported their location as “other specified” or “unspecified” or if their location was missing we treat this as time when children and parents were not at the same location.

The vast majority of total family time was at home (80.9% in 2000 and 79.6% in 2015). In both 2000 and 2015, around 8.5% of this time was spent traveling together (e.g., by car, walking, or bus), and the remainder was at other locations (e.g., at a restaurant or a sporting facility). We acknowledge the possibility of instances where children and parents were coded as being at the same location (e.g., being at a restaurant) at the same point in time on the same diary day but were in fact in different locations (e.g., different restaurants). However, given that the likelihood of this type of measurement error is negligible and does not vary systematically across the two surveys, it will not bias our comparisons over time.

We further decompose total family time into time when children reported being copresent with their parents as distinct from time when they were at the same location, but did not report being copresent with their parents. Although in both UKTUS surveys copresence was not restricted to being in the same room or engaging in the same activity, we argue that time when children do not report being copresent with parents more likely captures periods when children and parents are not closely interacting, perhaps being in separate rooms at home. Accordingly, we refer to these measures as copresent time and alone‐together time, respectively. The distinction between copresent and alone‐together time is not absolute, but children's reports of being copresent with parents does indicate an acknowledgement of awareness of being in close proximity to their parents notably absent during times when children choose not to report being copresent with parents despite being at the same location. We note that, in UKTUS 2000, children aged 14 to 16 years completed an adult diary and could therefore not indicate copresence with parents. We measure time with parents for these children as the time they reported being copresent with other household members (15 years and older) when they were at the same location as their parents. We tested the robustness of our results by excluding children aged 14 to 16 years living in households with others aged 15 years and older who are not their parents in both surveys, and the results remained the same.

To capture copresent time comprising relatively close interaction, we construct a further set of measures of the time children and parents spend engaging in shared activities using the main activity information provided by children and parents in their diaries. Specifically, we measure the total time in shared activities as well as measures of shared time in key activities around television viewing, eating, and other leisure during copresent time.

We note that, unlike existing family time measures based on parental copresence reports, our copresent time measure includes time when children are copresent with both parents (in two‐parent households) as well as time copresent with either their mother or their father, also known as “dyadic” time (Lam et al., 2012). In lone‐parent families, all family time based on copresence reports is dyadic, whereas there is no reason to ignore this time in two‐parent families. Indeed, there is an analog in parental measures of family time that do not distinguish between the number of children who parents are copresent with in multichild households. Our measure of copresent time is comparable with measures of the total time children are with their parents previously used in other studies (e.g., Sandberg & Hofferth, 2001) and overlaps substantially with measures of the total time that parents (individually) spend with children (Genadek et al., 2016; Neilson & Stanfors, 2017). In this study, however, copresent time forms part of an integrated set of measures relating to different aspects of the time children and parents are together, which we refer to jointly as measures of family time.

### *Measures of Device Use*

Our measure of digital device use comes from the additional device use column included in the UKTUS 2015 diary, providing a unique measure of concurrent use of digital devices during other daily activities (Hampton, 2017). Using these unique data, we decompose our abovementioned family time measures into time when children and parents were using digital devices and time when they were not using these devices in 2015. This permits us to explore the extent to which time using mobile devices overlaps with different types of family time capturing varying levels of interaction.

### *Analytical Technique*

The analysis is set out in the following two parts: The first part examines changes in different types of family time between 2000 and 2015, and the second part focuses on the embedding of mobile device use in family life in 2015. We use ordinary least squares for the multivariate analysis, which is appropriate for modeling time‐diary data (Stewart, 2013).

They key independent variable of interest in the first part of our analysis is survey year (2000 = 0 [reference]; 2015 = 1). Our analyses control for a number of factors potentially associated with family time as well as device use. As previously discussed, we expect to find more pronounced changes among older children given that earlier research shows that children spend less time with their parents in adolescence (Lam et al., 2012) and that mobile device ownership and use increases with age (Livingstone, 2002; Mullan, 2017). We focus on three age groups (8–10, 11–13, and 14–16 years of age) that correspond to distinctive schooling periods in the United Kingdom. We also adjust for sex differences in device use (Mullan, 2017). Socioeconomic differences are captured by a binary variable on household work status (jobless household: yes or no), and a binary variable on parental education (any parent has a degree or higher qualification: yes or no). Although socioeconomic status has generally been shown to make little difference in relation to adolescent mobile device use (Livingstone, 2002), it is associated with distinctive parental ideologies and attitudes toward mobile device use as well as working time patterns that may potentially impinge on different measures of family time (Chatzitheochari & Arber, 2012; Livingstone, 2007; Sayer, Gauthier, & Furstenberg, 2004). Family structure has clear implications for time allocation (Genadek et al., 2016) and is captured by the following two variables: lone‐parent household (yes or no) and number of children aged between 0 to 16 years. Last, we control for day type (weekday or weekend day) as families typically spend more time together on weekends than weekdays (Genadek et al., 2016). Table 1 provides descriptive statistics for all independent variables.

**Table 1:** Sample Characteristics

		Survey year	
Variables		2000	2015
	Number of diary days	3,182	1,811
		**%**	
Day type	Weekday	49.9	50.7
	Weekend	50.1	49.3
Age group	8–10 years	36.4	33.7
	11–13 years	34.5	32.1
	14–16 years	29.1	34.2
Sex	Boys	51.1	49.3
	Girls	48.9	50.7
Household employment status	Parent employed	83.2	87.4
	Jobless household	16.8	12.6
Parental education	No parent has degree	81.8	63.1
	Parent has degree	18.2	36.9
Household type	Two‐parent household	69.1	68.2
	Lone‐parent household	30.9	31.8
Number of children 0–16 years	Average (*SE*)	2.3 (0.02)	2.2 (0.02)

*Source*. U.K. Time Use Survey 2000 to 2001 and 2014 to 2015.

*Note*. Weights applied.

## Results

Table 2 presents descriptive statistics for the different types of family time. It shows that, in 2000, children spent 347 minutes per day at the same location as their parents and that approximately one third of this time (95 minutes) was alone‐together time. Although there was an increase in the total family time in 2015 (379 minutes), Table 2 shows that this was entirely driven by more alone‐together time (136 minutes). Last, Table 2 shows that children spent close to 84 minutes in 2000 and 87 minutes in 2015 in shared activities with their parents.

**Table 2:** Descriptive Statistics for Measures of Family Time

	Survey year	Average minutes	95% confidence interval	
Measures of family time			Minimum	Maximum
Total family time	2000	346.7	339.9	353.5
	2015	379.2	369.9	388.4
Copresent time	2000	252.1	245.1	259.1
	2015	243.3	234.4	252.2
Alone‐together time	2000	94.6	89.7	99.5
	2015	135.8	129.3	142.3
Shared activities, total time	2000	83.9	80.4	87.3
	2015	87.0	82.1	91.9
Eating	2000	15.8	14.9	16.6
	2015	20.1	18.8	21.4
Television	2000	33.5	31.6	35.4
	2015	27.2	24.8	29.6
Other leisure	2000	8.3	7.2	9.5
	2015	13.8	12.0	15.7

*Source*. U.K. Time Use Survey 2000 to 2001 and 2014 to 2015.

*Note*. *N* = 4,993. Weights applied.

Table 3 presents multivariate analysis results for three of our family time measures (total family time, copresent time, alone‐together time). It confirms that, controlling for other factors, there was no significant change in the time children were copresent with their parents between 2000 and 2015. However, alone‐together time increased significantly by 38.4 minutes. The overall increase in total family time was slightly less than half an hour. Further analysis (available upon request) showed that the increase in alone‐together time and total family time was comprised entirely of time spent at home.

**Table 3:** Ordinary Least Squares Estimates for Measures of Family Time: Copresent Time, Alone‐Together Time, and Total Family Time, Minutes per Day

	Models 1–3, Without Survey × Age interactions			Models 4–6, with Survey × Age interactions		
	Model 1	Model 2	Model 3	Model 4	Model 5	Model 6
Independent variables	Copresent time	Alone‐together	Total family time	Copresent time	Alone‐together	Total family time
2015 (ref. 2000)	−7.6	38.4***	30.9***	6.2	19.0*	25.2*
11–13 years (ref. 8–10 years)	−55.7***	16.1**	−39.5***	−50.0***	12.3	−37.7***
14–16 years (ref. 8–10 years)	−116.6***	21.4***	−95.1***	−107.1***	3.2	−103.9***
2015 × 11–13 years (ref. 2000, 8–10 years)	—	—	—	−16.5	11.3	−5.2
2015 × 14–16 years (ref. 2000, 8–10 years)	—	—	—	−25.5	48.3***	22.8
Girl (ref. boy)	−5.9	−6.1	−12.0*	−5.4	−6.7	−12.1*
Jobless household (ref. one or both parents in paid work)	5.3	28.0***	33.3***	5.2	29.1***	34.2***
Parent has degree (ref. neither parent has a degree)	6.5	14.1*	20.6**	5.9	15.5*	21.4**
Number of children 0–16 years (centered)	−5.6	6.7*	1.1	−5.7	6.9**	1.3
Lone‐parent household (ref. two‐parent household)	−66.3***	−26.2***	−92.5***	−66.3***	−26.4***	−92.7***
Weekend (ref. weekday)	104.0***	40.8***	144.8***	104.0***	40.8***	144.8***
Intercept	298.3***	77.4***	375.8***	293.6***	83.9***	377.5***
Adjusted *R* ^2^	0.13	0.05	0.20	0.13	0.05	0.20

*Source*. U.K. Time Use Survey 2000 to 2001 and 2014 to 2015.

*Note*. *N* = 4,993. Robust cluster standard errors adjust for multiple observations per person. ref. = reference.

* *p* < .05.

** *p* < .01.

*** *p* < .001.

The results for our controls were in line with existing time‐diary research (Table 3). Specifically, the models showed that all types of family time were substantially higher on weekend days and that children in single‐parent households spent less time with the coresident parent. Children in jobless households spent approximately 30 minutes more at the same location with their parents, but this was mostly alone‐together time. Similarly, parental education was positively associated with total family time and alone‐together time. We did not find significant differences by child sex. In contrast and according to our prior expectations, the results showed that the time children spent being copresent with parents decreased dramatically with age, accompanied by an increase in alone‐together time.

**Figure 1:**
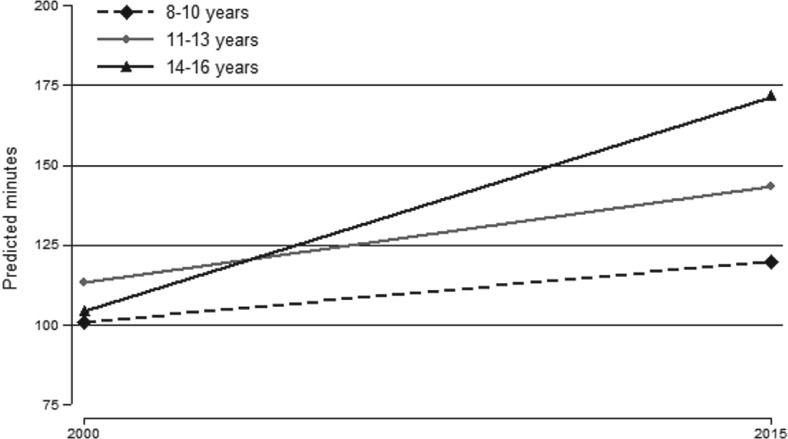
Predicted Minutes per Day Alone‐together by Child Age Groups and Survey Year.

We further tested whether changes in different types of family time, potentially associated with mobile device use, were consistent across children in different age groups, fitting an interaction between survey year and child age. These results are reported in Table 3 (M4–M6). We did not find significant interaction effects for copresent time, but we found a significant interaction effect for alone‐together time. In this interaction model (M5), the main effect for survey year remained significant but was substantially lower (19 minutes), whereas there was a significant positive interaction between survey year and age 14 to 16 years (48.3 minutes). To elaborate further on the substantive import of these results, Figure 1 shows the predicted minutes of alone‐together time by age group and survey year.

**Figure 2:**
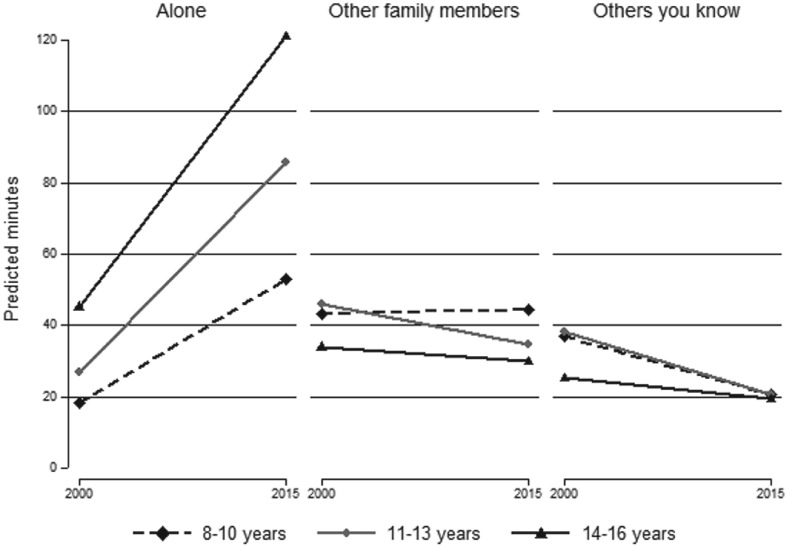
Predicted Minutes per Day Child is Alone, With Other Family Members, or With Others They Know during Alone‐together Time by Child Age Groups and Survey Year.

Figure 1 shows the predicted minutes of alone‐together time in 2000 and 2015 for the children in all age groups. Alone‐together time increased significantly from 101 minutes in 2000 to 120 minutes in 2015 for children aged 8 to 10 years, and from 113 minutes in 2000 to 143 minutes in 2015 for children aged 11 to 13 years. However, this dissimilar increase was not statistically significant (see corresponding interaction effect in M5, Table 3). In contrast, alone‐together time increased by 67 minutes for children aged 14 to 16 years from 104 minutes in 2000 to 171 minutes in 2015, and this was significantly greater than the increase for children aged 8 to 11 years (by approximately 48 minutes). An additional postregression Wald test comparing the change in alone‐together time for children aged 11 to 13 years and 14 to 16 years showed that they were significantly different, *F*(1, 1866) = 7.72; *p* < .01), indicating that the increase in alone‐together time for children aged 14 to 16 years was significantly greater than the increase for children aged 11 to 13 years. To probe this trend further, we decomposed alone‐together time using information children provided about whom they were with (“alone,” with “other family members,” or with “others you know”). The descriptive results from this analysis are shown in Figure 2 (complete regression results are available on request).

The time children reported being “alone” when at the same location as their parents increased significantly for all children between 2000 and 2015 (Figure 2). However, increases for children aged 11 to 13 years (59 minutes) and for children aged 14 to 16 years (76 minutes) were greater than for children aged 8 to 10 years (35 minutes). The difference in the change for children aged 11 to 13 years and 14 to 16 years (17 minutes) was only marginally statistically significant, *F*(1, 1866) = 3.21; *p* = .07. Time with other family members (e.g., siblings) did not change for children in the youngest or oldest age groups, but it decreased by 13.5 minutes for children aged 11 to 13 years. At the same time, the time children spent with others they know (e.g., friends) significantly decreased in all age groups.

We now turn to the regression results for time in shared family activities, reported in Table 4. The results showed that the total time family members spent doing activities together did not change significantly between 2000 and 2015. With regard to our activities of interest, we found increases in eating (4.2 minutes) and other leisure activities (5.5 minutes), and a decrease in television viewing (−4.1 minutes). This suggested that, despite ongoing concerns about the influence of mobile device use on family life, the amount of time families with children aged 8 to 16 years in the United Kingdom spend on shared activities has remained overall largely unchanged.

**Table 4:** Ordinary Least Squares Estimates for Shared Activity Measures: Total Time in Shared Activities, Total Shared Time Eating, Total Time Television Viewing, and Total Time in Other Leisure Activities, Minutes per Day

Independent variables	Total time in shared activities	Shared time eating	Shared time television viewing	Shared time in other leisure
2015 (ref. 2000)	6.4	4.2***	−4.1*	5.5***
11–13 years (ref. 8–10 years)	−19.0***	−4.9***	1.4	−2.9
14–16 years (ref. 8–10 years)	−32.6***	−6.3***	0.6	−8.3***
Girl (ref. boy)	3.5	−0.8	−0.2	0.1
Jobless household (ref. one or both parents in paid work)	3.0	2.5*	4.4	1.4
Parent has degree (ref. neither parent has a degree)	3.1	5.4***	−9.0***	0.4
Number of children 0–16 years (centered)	−7.1***	−1.6***	−2.2*	−1.5*
Lone‐parent household (ref. two‐parent household)	−38.1***	−8.2***	−14.3***	−6.1***
Weekend (ref. weekday)	44.7***	7.9***	11.6***	12.7***
Intercept	93.8***	17.9***	33.6***	9.8***
Adjusted *R* ^2^	0.09	0.07	0.03	0.04

*Source*. U.K. Time Use Survey 2000 to 2001 and 2014 to 2015.

*Note*. *N* = 4,993. Robust cluster standard errors adjust for multiple observations per person. ref. = reference.

* *p* < .05.

*** *p* < .001.

There is no prior research on changes in time in shared activities, so the results for our control variables provided some valuable insights in this regard. Table 4 shows that time in shared family activities decreased with age and that there were no gender differences. There were no differences in shared activities by household employment status or parental education either. However, children with a parent with a degree spent less time in shared television and more time in shared eating. Time in shared activities was negatively associated with the number of children in the family, perhaps reflecting difficulties of larger families to coordinate shared activities. As with other measures of family time, time in shared activities was lower in lone‐parent families and higher on weekends.

### *Family Time and Mobile Device Use*

We now proceed to the analysis of the time children and parents spend using mobile devices using data collected in UKTUS 2015. Table 5 provides a descriptive analysis of the average minutes per day children and parents, individually and combined, spent using digital devices by different types of family time. To assess the relative concentration of time using devices during different aspects of family time, the first column of Table 5 reports the average time for each measure of family time in total.

**Table 5:** Mean Mobile Device Use During Different Types of Family Time, Children and Parents, Minutes per Day

Measures of family time	Total	Child using device	Parent using device	Child or parent using device
Total family time	381.7	87.2	83.4	145.7
Copresent time	245.1	43.5	48.7	80.7
Alone‐together time	136.5	43.7	34.7	65.0
Shared activities				
All shared activities	88.1	11.2	16.9	24.5
Eating	20.3	1.5	3.1	4.2
Television viewing	27.6	5.2	6.9	10.5
Other leisure	14.1	2.1	2.8	3.9

*Source*. U.K. Time Use Survey 2014 to 2015.

*Note*. *N* = 1,775. Weights applied.

Table 5 shows that, in 2015, children were using a mobile device during 87 minutes of total family time, which represents about one fifth of this time. This was split almost equally between copresent (43.5 minutes) and alone‐together time (43.7 minutes). However, children spent proportionally more time using devices during alone‐together time (32%) than during copresent time (18%). Table 5 also shows that parents had a similar average of device use across total family time (83.4 minutes), whereas the average time either children or parents used a device during total family time was 145.7 minutes (38% of total family time). Similarly, parental device use was more concentrated during alone‐together time, with either parents or children spending approximately half (48%) of this time using a mobile device compared with around one third of all copresent time.

Both children and parents used devices when engaging in shared activities, although this was marginally higher among parents than children (Table 5). Overall, during time in shared activities, children spent about 11 minutes using a device (13% of the total), whereas parents spent 16.9 minutes (19% of the total). This difference was particularly stark when examining family meals. Bearing in mind that the absolute amounts were small (3.1 and 1.5 minutes respectively), in relative terms parents reported using a device for 15% of all time in shared eating compared to 7% for children. Device use was mostly concentrated during television viewing, with either parents or children reporting using a device for close to 40% of this time.

To further understand the relationship between device use and family time, we  estimated models of family time (time copresent, alone‐together time, and total time in shared activities) distinguishing between time when children reported using a mobile device and time when they did not. Table 6 demonstrates the results of these models.

**Table 6:** Coefficients From Models of Family Time With and Without Device Use in 2015, Minutes per Day

	Copresent time, child using device		Alone‐together time, child using device		Shared activities, child using device	
Independent variables	No	Yes	No	Yes	No	Yes
11–13 years (ref. 8–10 years)	−78.0***	10.8*	10.9	16.1***	−29.3***	4.6*
14–16 years (ref. 8–10 years)	−148.6***	16.6**	5.1	44.4***	−55.8***	8.6***
Girl (ref. boy)	−2.0	−10.7*	1.0	−14.4**	−1.7	−1.3
Jobless household (ref. one or both parents in paid work)	−1.5	−2.5	16.3	11.2	2.4	−4.1
Parent has degree (ref. neither parent has a degree)	3.5	−7.9	6.8	−3.4	0.7	−1.4
Number of children 0–16 years (centered)	6.2	−5.7*	7.5*	−5.8*	−4.3	−1.8
Lone‐parent household (ref. two‐parent household)	−59.3***	−1.2	−15.0*	−7.7	−36.9***	−2.3
Weekend (ref. weekday)	88.0***	14.2***	27.3***	17.4***	49.9***	6.2***
Intercept	269.0***	40.3***	80.1***	27.8***	100.7***	7.1***
Adjusted *R* ^2^	0.20	0.02	0.02	0.07	0.13	0.02

*Source*. U.K. Time Use Survey 2014 to 2015.

*Note*. *N* = 1,775. Reference category is children aged 8–10 years. ref. = reference.

* *p* < .05.

** *p* < .01.

*** *p* < .001.

Table 3 earlier showed that copresent time was negatively associated with age. Table 6 shows that, in 2015, copresent time was overwhelmingly concentrated in time when children were not using a device. In fact, time using a device during copresent time increased with age, being significantly higher for children aged 14 to 16 years compared with children aged 8 to 10 years. In other words, older children spent less time copresent with their parents, although more of their remaining time copresent with parents co‐occurred with time they spent using devices. Turning to the results from the models of alone‐together time, these showed that the positive effect associated with children's age was entirely concentrated in time when children were using devices. The results were particularly striking for children aged 14 to 16, as nearly all of the increase in alone‐together time was concentrated in time when they were using devices. The results for time in shared activities were substantively similar to those for overall time copresent with parents. We also estimated models for measures of family time when parents reported using devices or did not. The results (available upon request) showed that the effects of child age groups for each aspect of family time were similar irrespective of whether a parent reported using a device or not.

## Discussion and Concluding Remarks

The past decade has witnessed increasing concerns surrounding the impact of the diffusion of Internet‐enabled mobile devices on individual and family well‐being (Gergen, 2002; Palmer, 2015; Turkle, 2011; Twenge, 2017). However, nationally representative research on the impact of mobile device use within the family context remains scarce. This article sought to rectify this by providing novel evidence from time‐use surveys on the ways family time has changed during a period of unprecedented technological change in the United Kingdom. Grounded in theoretical perspectives on the influence of recent technological change on family life, we developed a rich set of family time measures capturing different degrees of togetherness and interaction of children and parents. This allowed us to move beyond existing time‐diary analyses on family time trends that do not sufficiently capture aspects of family time most likely influenced by mobile devices (Genadek et al., 2016; Neilson & Stanfors, 2017; Sandberg & Hofferth, 2001). In addition to this, we produced a novel account of the embedding of mobile device technology on different types of family time and activities in 2015, complementing existing qualitative studies on the same topic (Bond, 2014; Clark, 2013; Ling, 2012). An additional contribution of our work is that, by centering our analytical approach on children's diary reports, we were able to include both two‐parent and lone‐parent families in our analysis of family time trends.

Our results show an increase in the time children and parents spent together at the same location. We found that this increase was overwhelmingly composed of more time spent at home, which is consistent with earlier theoretical accounts and empirical evidence around the potential of technology to enhance the home environment and bring family members together (Daly, 1996; Ogburn & Nimkoff, 1955; Rainie & Wellman, 2012). These results provide further support for the argument that changes in mobile technologies have led to children and young people increasingly spending time at home (Twenge, 2017). These findings also broadly align with recent time‐diary research showing increases in the time parents are copresent with children (Genadek et al., 2016, Neilson & Stanfors, 2017). However, decomposing this time showed that this increase did not stem from an increase of copresent time, that is, time of closer interaction of family members. In fact, we found that copresent time has remained constant between 2000 and 2015, whereas alone‐together time has significantly increased. Our finding that children explicitly reported being “alone” during alone‐together time sheds further light into the nature of this type of time, whereas our finding that device use was also disproportionately concentrated within alone‐together time strengthens our claim about an association with recent technological change.

Our analysis also showed that the increase of alone‐together time was overwhelmingly concentrated among children aged 14 to 16 years. We might interpret this as supporting evidence of a causal relationship of mobile device use with alone‐together time, considering that children's use of these technologies increases with age (Mullan, 2017). However, we note that middle and late adolescence also constitute a developmental stage whereby children become more autonomous, spending less time with their parents and more time with their friends (Steinberg, 2002). Our results show that, in the case of Britain, older children are increasingly carving out “alone” time while being at the same location with their parents. In relation to this, we also note persistent concerns about safety outside the home in the United Kingdom (Shaw et al., 2013) that may be contributing to these increases in time children are at home with their parents in tandem with alone‐together time within the home. It is unclear, therefore, whether adolescents are increasingly “kept” at home due to safety concerns or whether technological change and the entertainment and communication opportunities it provides have been driving these trends.

Although the results around alone‐together time provide evidence of change in family time in the United Kingdom in the past decade, our data also show evidence of stability in other dimensions of family time. In addition to the abovementioned lack of change in copresent time, we also find little evidence of change in the total time children and parents spent doing shared activities. In accordance with existing research on changing television viewing practices (Ofcom, 2015), our data suggest that shared television viewing somewhat decreased in the past 15 years in the United Kingdom. This may be partly driven by the decrease of television viewing among children (Mullan, 2017). However, small increases in family meals and other shared leisure activities offset the decrease in television viewing, and the total time children and parents spent doing shared activities remained unchanged. These findings accord with sociological accounts about parents valorizing and protecting some key daily family practices (Daly, 2001), which appear unaffected by technological change. They also highlight how increases in the time parents report being copresent with children found in previous research (Genadek et al., 2016, Neilson & Stanfors, 2017) might not necessarily entail more “quality” time in shared activities.

Decomposing time at the same location into copresent and alone‐together time allowed us to shed light into the changes in the nature of family time, which was further complemented by the examination of data on mobile device use in 2015. Corroborating qualitative studies, this part of our analysis showed that device use overlaps with all types of family time, including alone‐together time and time of closer interactions such as shared activities. We have already noted that the disproportionate overlap with alone‐together time shows how device use lends itself to relatively individualized time. Overlaps with time copresent and time in shared activities point to a further set of issues encapsulated in various theoretical arguments around states of absent presence (Gergen, 2002) and alone‐together (Turkle, 2011), whereby mobile devices are argued to distract people's attention during different types of family time and activities. This is another dimension of alone‐together time, which has attracted most attention recently, and is particularly tied to mobile device use.

The use of devices when copresent and in shared activities has implications for the quality and overall experience of family time and may even explain parents' perceptions of decreasing family cohesion and decreased time together with children, as reported in earlier studies (Mesch, 2006; Williams & Merten, 2011). Indeed, research has established that the use of mobile devices significantly decreases the quality of face‐to‐face interactions (Kildare & Middlemiss, 2017; Misra et al., 2016; Przybylski & Weinstein, 2013; Thornton et al., 2014).

Our findings provide a first indication of an upper limit on the amount of family time that might be “contaminated” or in some way eroded by device use. It is an open question as to whether this is “too much” or whether it represents a substantial negation of the quality of family time. Although not addressing this question, our results offer a kind of baseline assessment of the extent to which device use overlaps with different types of family time, which should be monitored over time and that can be compared across different countries. We hasten to add that it is not the only indicator and that it should be supplemented with other indicators. However, we argue that knowing broadly how much time is “affected” by mobile device use nonetheless provides useful perspective for debates in this area.

We note that our analysis presents a number of limitations, particularly in relation to gauging whether mobile devices are affecting experiences of family time. First, time‐diary data do not provide any information about the content of device use, and we are therefore not able to say whether mobile device use is interrupting or complementing family interactions. In a similar manner, we note that mobile device use has been shown to aid children and young people in forming networks and maintaining friendships outside the home (Bond, 2014; Clark, 2013). This is an important note to make as it suggests that mobile device use may be a positive influence for some relationships at the expense of others. Second, although time‐diary data allow us to construct a wide range of objective measures of family time and activities, we did not have information on subjective experiences of time in UKTUS 2000. This would enable us to examine subjective feelings during different types of family time including alone‐together time. Enjoyment data from UKTUS 2015 could be employed to better understand the associations of mobile device use with subjective experiences of family time of children and parents. Third, similar to earlier research on family time, we abstracted from possible differences between children's time with mothers, fathers, or with both. Although our aim was to provide a broad picture in trends on different types of family time, future research could delve further into these differences to better understand the ways different family relationships and family practices are affected by mobile device use.

Notwithstanding these limitations, this article sheds light on changes in different aspects of family time and on the extent to which mobile devices are now embedded in the time parents and children spend together. In understanding the influence of mobile devices on family time, it is necessary to consider the spatial and interactional dimensions of family life. In doing so we have demonstrated that a simple one‐dimensional view of family time is insufficient for understanding the potential influence of mobile devices and that relatively recent concerns about technological changes possibly eroding family interactions cannot easily be separated from a long‐standing tension surrounding the influence of technology as bringing family members together while keeping them apart. Our findings suggest moving beyond a sole concern with the amount of time children (and parents) spend using mobile devices, drawing attention to a critical intersection between family time, mobile device use, and adolescent development.

## Note

This research was based on the United Kingdom 2000 to 2001 Time Use Survey, produced by the Office for National Statistics and IPSOS‐RLS, and the 2014–2015 Time Use Survey, produced by the National Centre for Social Research and the Northern Ireland Statistics and Research Agency on behalf of the University of Oxford. Data are Crown Copyright and were supplied by the U.K. Data Service, which bears no responsibility for any of the analyses and interpretations presented in this article. Killian Mullan's contribution was supported by the Economic and Social Research Council (ES/L011662/1) and by the Euopean Research Council (Project 339703).
